# Automated sleep stage scoring employing a reasoning mechanism and evaluation of its explainability

**DOI:** 10.1038/s41598-022-16334-9

**Published:** 2022-07-27

**Authors:** Kazumasa Horie, Leo Ota, Ryusuke Miyamoto, Takashi Abe, Yoko Suzuki, Fusae Kawana, Toshio Kokubo, Masashi Yanagisawa, Hiroyuki Kitagawa

**Affiliations:** 1 Center for Computational Sciences, University of Tsukuba, Tsukuba, Japan; 2 International Institute for Integrative Sleep Medicine (WPI-IIIS), University of Tsukuba, Tsukuba, Japan; 3 Yumino Heart Clinic, Toshima, Japan; 4 Juntendo University Graduate School of Medicine, Bunkyo, Japan; 5 S’UIMIN Inc., Shibuya, Japan; 6 R&D Center for Frontiers of Mirai in Policy and Technology, University of Tsukuba, Tsukuba, Japan; 7 Tsukuba Advanced Research Alliance (TARA), University of Tsukuba, Tsukuba, Japan; 8 Department of Molecular Genetics, University of Texas Southwestern Medical Center, Dallas, USA

**Keywords:** Electrodiagnosis, Computational science

## Abstract

Scoring sleep stages from biological signals is an essential but labor-intensive inspection for sleep diagnosis. The existing automated scoring methods have achieved high accuracy but are not widely applied in clinical practice. In our understanding, the existing methods have failed to establish the trust of sleep experts (e.g., physicians and clinical technologists) due to a lack of ability to explain the evidences/clues for scoring. In this study, we developed a deep-learning-based scoring model with a reasoning mechanism called class activation mapping (CAM) to solve this problem. This mechanism explicitly shows which portions of the signals support our model’s sleep stage decision, and we verified that these portions overlap with the “characteristic waves,” which are evidences/clues used in the manual scoring process. In exchange for the acquisition of explainability, employing CAM makes it difficult to follow some scoring rules. Although we concerned the negative effect of CAM on the scoring accuracy, we have found that the impact is limited. The evaluation experiment shows that the proposed model achieved a scoring accuracy of $86.9\%$. It is superior to those of some existing methods and the inter-rater reliability among the sleep experts. These results suggest that Sleep-CAM achieved both explainability and required scoring accuracy for practical usage.

## Introduction

Mammalians’ sleep consists of three main stages (states), namely, wake (awake/drowsy), REM (rapid eye movement) sleep, and non-REM sleep (it can be classified into three categories according to the depth of sleep^1^). These sleep stages play different roles in humans’ sleep and can be identified from biological signals.

Identification (scoring) of the sleep stage is essential for sleep medicine. The distribution and transition of sleep stages suggest the quality of the patient’s daily sleep and the existence of sleep disorders. For example, sleep-deprived individuals tend to fall into a deep sleep immediately after lying down on the bed. Furthermore, abnormal sleep stages correlate with many diseases, such as narcolepsy, parasomnia, and insomnia.

Unfortunately, manual sleep-stage scoring is a very labor-intensive task. Sleep experts (e.g., physicians and clinical technologists) need to visually detect the “characteristic waves” of sleep stages, i.e., the waveforms related to a particular sleep stage^1^. This process forces the experts to spend a considerable amount of time and to have a high level of expertise. Consequently, a clinically applicable automated sleep-stage scoring method is currently in high demand by professionals who want to improve/facilitate sleep medicine.

To fulfill this demand, many researchers have proposed automated scoring methods that achieve scoring accuracy (i.e., agreement rate with sleep experts) high enough for clinical usage^2–13^. However, these methods have rarely been used in clinical practice.

In our understanding, these existing models have failed to gain credence from experts due to a lack of explanation of scoring evidences/clues. The sleep-stage scoring is a complex inspection, and the scoring results often differ depending on the expert. According to Danker-Hopfe, H. et al., the inter-rater reliability of sleep-stage scoring is $82.0\%$^25^. To reduce the scoring differences, the experts check their decisions with each other to see any discrepancy in the scoring criteria. They are asked to show the scoring evidences/clues to identify the sleep stages.

In contrast, most existing automated scoring methods cannot show these evidences/clues due to a lack of reasoning mechanisms^2–12^. So, the experts cannot understand the criteria for the scoring process. The users cannot even know what characteristic waves were focused/detected. It prevents the experts from relying on the scoring methods.

To develop a clinically applicable scoring method, we designed a novel deep-learning-based model, Sleep-CAM, with a reasoning mechanism showing the intervals that work as evidences and clues in the stage assignment. The reasoning mechanism helps the experts understand whether the scoring process is logical and reasonable. It will establish credence from the experts and facilitate the double-checking process by showing which sub-intervals should be double-checked manually. Besides, the Sleep-CAM employs the same set of biological signals as the manual scoring process. Using the same type/number of biological signals guarantees the systems analyze the same information as the experts.

To achieve such explainability, we employed convolutional neural networks (CNNs) and class activation mapping (CAM)^18^. CNNs are known to be excellent models that can locate useful features for classification tasks^14–17^. For example, many electrocardiogram analysis systems employ CNNs to realize accurate atrial fibrillation and arrhythmia detection^14,15^. This type of neural network is suitable for detecting the occurrences of characteristic waves (Table 1)^1^. Therefore, CNNs are suitable for characteristic wave detection.

**Table 1:** Characteristic waves^1^.

Signal	Wave	Characteristics	Related stage
EEG	Alpha rhythm wave	An 8–13-Hz-frequency wave that mainly appears in occipital EEG results	W
	Arousal	An event where the frequency of EEG signals suddenly change. After this event, sleep shifts to a lighter stage, i.e., W or N1. Additionally, EMG activity related to body movement often appears at this time	W & N1
	Low-amplitude mixed-frequency activity	A low-amplitude, 4–7-Hz-frequency wave	N1 & REM
	Vertex wave	A sharp wave with a duration of less than 0.5 s. This wave can be clearly distinguished from background EEG signals	N1
	K-complex	A clear negative-sharp wave that is immediately followed by a positive wave. This wave can be clearly distinguished from background EEG signals. Its duration exceeds 0.5 s	N2
	Sleep spindle	A clear wave with a frequency of 11–16 Hz, and duration of more than 0.5 s. The maximum amplitude is located at the center of the wave. This wave can be clearly distinguished from background EEG signals	N2
	Delta rhythm wave	A wave with a frequency of 0.2–5 Hz and peak amplitude that exceeds 75 $\mu {\mathrm{V}}$. It often appears in frontal EEG	N3
	Saw-tooth wave	A saw-tooth wave with a frequency of 2–6 Hz	REM
EOG	Blink	Vertical eye movement with a frequency of 0.5–2 Hz	W
	Slow-eye movement	Regular and sinusoidal eye movement	W & N1
	Rapid-eye movement	Random and rapid eye movement	W & REM
EMG	Weakness	The tension becomes decreases to less than that in other sleep stages	REM
	Temporal contraction	Irregular bursts of myoelectrical activity	REM

Note that the AASM rule defines arousal as a sleep event, not a characteristic wave.

However, we have decided to treat arousal as a type of characteristic wave in this study.

CAM^18^ is a reasoning mechanism for deep-learning models that entail the inclusion of a global average pooling (GAP) layer^19^. First, our model estimates a class activation map, which shows each stage’s likelihood at each time point. Then, it aggregates the results by taking the average of the likelihood values along the time axis. The values of CAM will be optimized through the training process. The loss will be backpropagated more to the intervals with waveforms that often appear in specific sleep stages. Therefore, the CAM values in the interval with such waves will be higher than the others. This difference suggests which portions of the PSG signals are more contributing to scoring sleep stages.

Although the GAP layer is essential to achieve explainability, it will prevent the Sleep-CAM from following some scoring rules^1^. For example, our proposed model cannot consider when the characteristic waves appeared because the GAP layer integrates the likelihood values across the time axis.

To verify the impact of the GAP layer and efficacy/accuracy of Sleep-CAM, we conducted scoring accuracy evaluation experiments using 109 whole-night sleep records. The experimental results show that Sleep-CAM achieved a scoring accuracy of $86.9\%$, higher than the inter-rater reliability among the experts and some existing automated methods. It suggests that Sleep-CAM provide scoring accuracy required for practical usage.

## Sleep stage scoring

The American Academy of Sleep Medicine (AASM) defines five stages of human sleep: W (awake or drowsy), REM (REM sleep), and N1, N2, and N3 (non-REM sleep)^1^. Note that there are three sleep stages in non-REM sleep. They differ in the depth of sleep, as the sleep deepens from stage N1 to N3.

Manual sleep-stage scoring requires the following four steps.

1. PSG signal acquisition: The experts measure six electroencephalogram (EEG) signals, two electrooculogram (EOG) signals, and one submental electromyogram (EMG) signal from the patient. Characteristic waves in the EEG signals may appear clearly in some measurement channels but not so clearly in other channels; therefore, measuring EEG signals from multiple channels is necessary for accurate characteristic wave detection.
2. Preprocessing: The measured PSG signals are often contaminated by noise caused by motion artifacts, electrode popping, and the power supply. The experts use frequency filters to eliminate these noises. After noise elimination, the signals are divided into 30-s sub-sequences called epochs. An epoch is a unit of time for scoring, and one sleep-stage label will be assigned to each epoch.
3. Characteristic-wave detection: The experts detect the characteristic waves from the PSG signals. The details of each characteristic wave are described in the next chapter.
4. Sleep stage assignment: The experts assign one sleep stage according to the AASM rule. This rule mainly follows the following three principles:
  If the epoch, especially the first half, contains the characteristic waves, it will be scored as the sleep stage associated with these waves.
  If the epoch has no characteristic waves, it will be scored considering the stage and the characteristic waves of the preceding epochs.
  If the epoch contains multiple characteristic waves associated with different sleep stages, the epoch will be scored as the dominant sleep stage.

### Characteristic waves

The AASM defines 12 characteristic waves for sleep-stage scoring (Table 1), and each characteristic wave is associated with one or two sleep stages. For example, the alpha rhythm wave, i.e., an 8–13-Hz-frequency wave, only appears in Stage W; therefore, the experts can use these signals as the reason for the Stage W assignment. The scoring results are significantly dependent on the characteristic waves; thus, accurate characteristic wave detection is essential for accurate sleep-stage scoring.

Note that the significance of each type of characteristic wave is different. For instance, experts tend to value rapid-eye-movement waves in EOG signals more than EMG weakness.

Unfortunately, it is challenging to develop characteristic wave detection methods. There are many types of characteristic waves, and each of them is very sensitive to noise and individual differences. In addition, both the frequency-domain and time-domain features must be considered to detect these characteristic waves. These facts make it difficult to develop an effective feature-detection method manually.

## Results

Here, we introduce our proposed method, Sleep-CAM, and evaluate its explainability by analyzing the relationship between focused intervals in the automated scoring process and the occurrence of the characteristic waves. In addition, we also verify the scoring performance of Sleep-CAM through some experiments including the comparison with existing models.

### Sleep-CAM

Sleep-CAM is a deep-learning model for characteristic-wave detection and stage assignments for sleep-stage scoring. This model also has a reasoning mechanism that distinguishes the intervals that work as evidences/clues in stage assignments.

**Figure 1:**
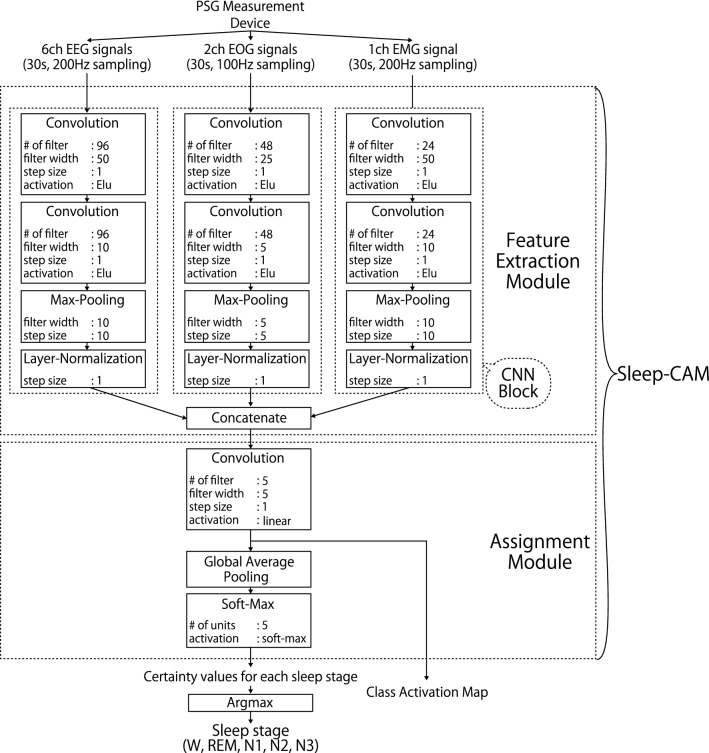
Structure of Sleep-CAM. Sleep-CAM has six types of layers: convolution, max-pooling, layer-normalization, concatenate, global average pooling, and softmax layers. The hyper-parameters of each layer are summarized in the boxes, and have been optimized through some preliminary experiments (Supplementary information).

In general, Sleep-CAM consists of two modules that are respectively associated with feature extraction and stage assignment steps (Fig. 1). The feature extraction module locates the useful features in the biological signals and extracts them for the assignment phase. The assignment module models the relationship between the extracted features and sleep-stage labels and estimates the label based on this relationship. The extracted features are expected to be correlated with the characteristic waves in manual scoring and still achieve considerably accurate sleep-stage assignments, as shown later.

One major characteristic is that the proposed model considers only the PSG signals in the target epoch. As aforementioned, the experts consider not only the characteristic wave in the target epoch but also the stage transition and stage tendencies of surrounding epochs. So, this characteristic may affect accuracy improvement. However, the main focus of this paper is to develop a reasoning mechanism and verify the relationship between the reasoning results and characteristic waves rather than aiming at improving the accuracy of the automated scoring systems. So, we prioritized making the reasoning result directly reflect the characteristic waves in the target epoch in this paper.

In this section, we discuss the details of the proposed model, which will be subsequently evaluated through experimental analysis. Note that the input is a PSG signal of one 30-s epoch, and the output is the sleep-stage label and the CAM. These signals were normalized such that their mean value and standard deviations were respectively equal to 0 and 1. We also assumed that the sampling frequencies of the EEG, EOG, and EMG signals were 200, 100, and 200 Hz, respectively. All parameters were optimized experimentally to suit the PSG signals of this measurement setup.

#### Feature extraction module

The feature extraction module locates the features of the PSG signals that are suitable for estimating the sleep-stage labels and extracts them for the scoring process. That is, this module conducts a similar process to the characteristic-wave detection in manual scoring.

This module consists of three CNN blocks that are each related to EEGs, EOGs, and an EMG, respectively. According to the AASM rules, there are no characteristic waves associated with multiple types of biological signals; therefore, we decided to employ a different CNN block for each type of signal. Although these blocks have the same structure, the hyper-parameters differ according to the sampling frequency and contribution to sleep-stage scoring. As an example, the number of associated characteristic waves is a factor for deciding the number of kernels. Each CNN block is composed of two convolutional layers, one max-pooling layer and one layer-normalization layer.

The convolutional layers quantify the convolution between the input signals and their respective kernels, which act as a type of short-width adaptive filter. Then, the results will be activated by the exponential linear unit (elu) function. These layers are primarily responsible for determining useful feature values and are expected to detect the characteristic waves. In contrast to the convolutional layers, the max-pooling layers are not trainable. It divides the inputted time-series data of extracted features into a plurality of sub-sequences and takes the maximum value in each sub-sequence. This process works to enhance the robustness against the time lag and to reduce computing time.

One of the characteristics of our model is to employ layer-normalization layers. In general, the batch-normalization layers are often employed to suppress the adverse effects related to differences in the distribution of samples and feature values; however, we found that the layer-normalization is more effective in the automated sleep-stage-scoring task. The layer-normalization layer normalizes the feature values across the “kernel” axis; so, the normalized values will be relative values of each feature at a particular point in time. Please see also the “Efficacy of layer normalization” section for the detail.

#### Assignment module

The assignment module models the relationship between the extracted feature values and the sleep-stage labels and estimates the latter from the former. Besides, it also outputs the CAM showing the likelihood of each sleep stage at each point in time. The key structure is the first convolutional layer and the following global-average-pooling (GAP) layer (Fig. 1). The convolutional layer has five kernels associated with each sleep stage and calculates the CAM. As mentioned above, these CAM values are used to illustrate the interval that forms the basis of the stage assignment.

Then, the GAP layer integrates the likelihoods by taking the average across the time axis. The integrated likelihoods will be converted into the sleep-stage confidence values by the soft-max activation. Actually, the operation of the GAP layer is a little different from that in the typical manual scoring. The experts consider when the characteristic waves appear and what percentage of each sleep stage is in the target epoch. However, the information on the time axis will be spoiled by the GAP layer. This seems to decrease the scoring accuracy. Contrary to this concern, we found that the GAP layer makes the training process easy and improve the scoring accuracy. Please see also the “Efficacy of the global average pooling” section for the detail.

#### Training

We employed an end-to-end training approach for Sleep-CAM; i.e., the model receives pairs of one-epoch PSG signals and sleep-stage labels assigned by an expert and will be optimized to score as close as the expert’s ground truth labels. Although we focus on the characteristic waves in this study, we did not use any information of characteristic wave occurrences/appearances in the training process. Sleep-CAM is expected to locate the effective features, such as characteristic waves, only from the information of sleep-stage label for the scoring epoch.

In this study, we fitted Sleep-CAM using the Adam method^20^ (${\mathrm{lr}} = 5\times 10^{-6}$) and a cross-entropy loss function. In addition, an early-stopping process has been implemented to avoid overfitting. The training process is terminated if the number of training iterations exceeds 200 or if the minimum value of validation error has not been updated in the last five iterations. $16.6\%$ (= 1/6) of the training samples (epochs) were firstly reserved and were used to calculate validation error.

### Explainability verification

As aforementioned, our proposed model has a reasoning mechanism that determines which sub-interval becomes the focus of the model in the scoring process. Here, we verified its explainability through the analysis of the model used in the evaluation experiment. Please see the next chapter for the details of the training setting and datasets.

**Figure 2:**
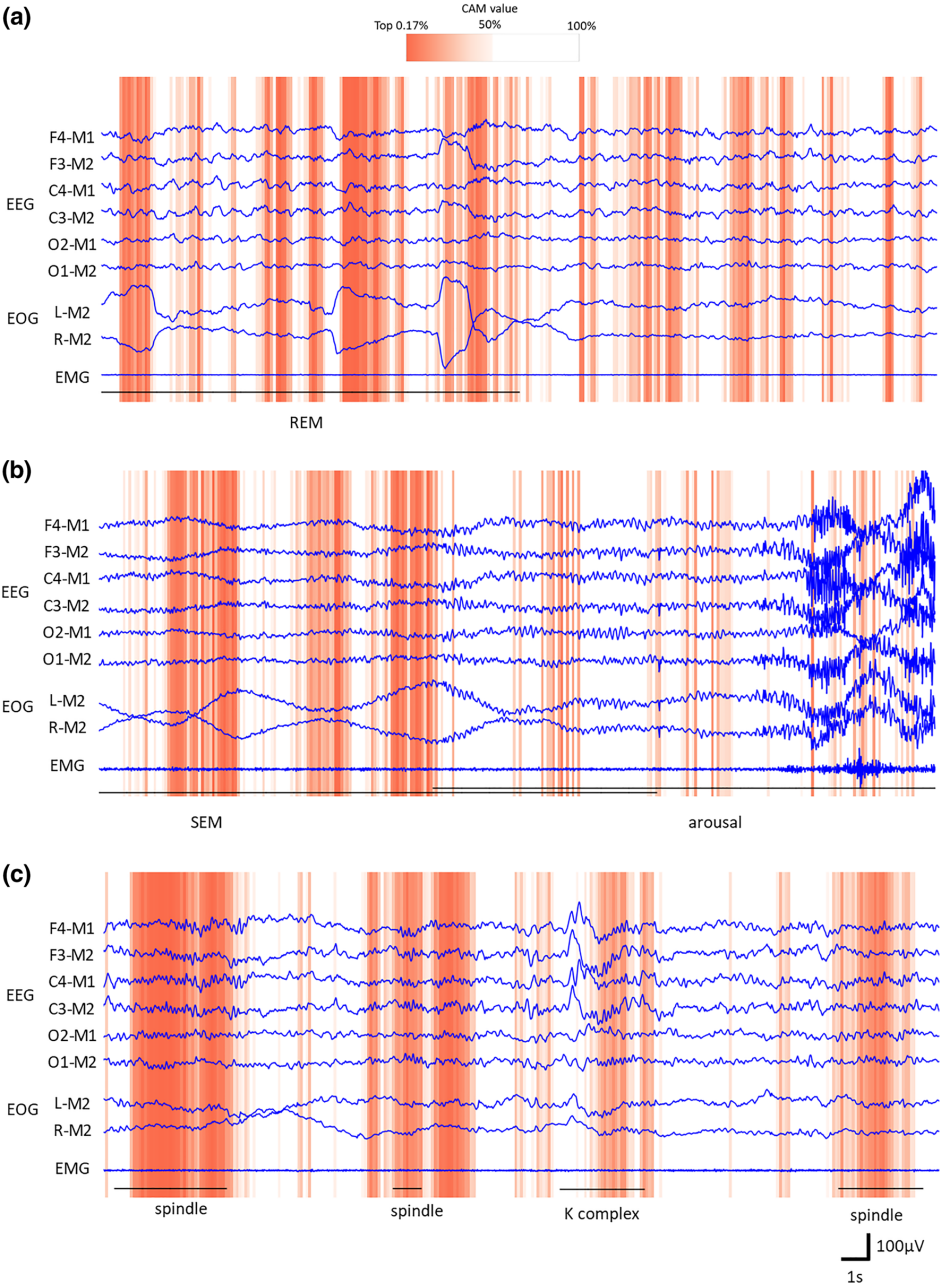
Examples illustrating the reasoning results. The sub-intervals with darker-orange background had high CAM values and were more focused in the scoring process. The black line shows the width of the characteristic waves the experts designated.

Figure 2 provides examples, the epoch of Stages REM, N1, and N2. The sub-intervals that the model focused on are illustrated with the dark-orange background. Besides, the characteristic waves detected by an expert are shown with the black line and its name. In many cases, the Sleep-CAM focused on convincing sub-intervals. For example, Fig. 2a indicates the Sleep-CAM assigned higher CAM values to the first half of the epoch. There is a REM wave (rapid change of the EOG) that is an important clue to score Stage REM. In the case of Fig. 2b, you can see that the model extracts a part of the SEM. Concretely, it seems to extract the left-side EOG signal rises. Besides, this figure also indicates that Sleep-CAM considers the epoch with alpha waves to be not Stage N1. Of course, several types of characteristic waves are difficult to detect. Especially, the K-complex waves regarding Stage N2 were often overlooked (Fig. 2c). Here, let us show what characteristic waves were extracted in the scoring process.

**Figure 3:**
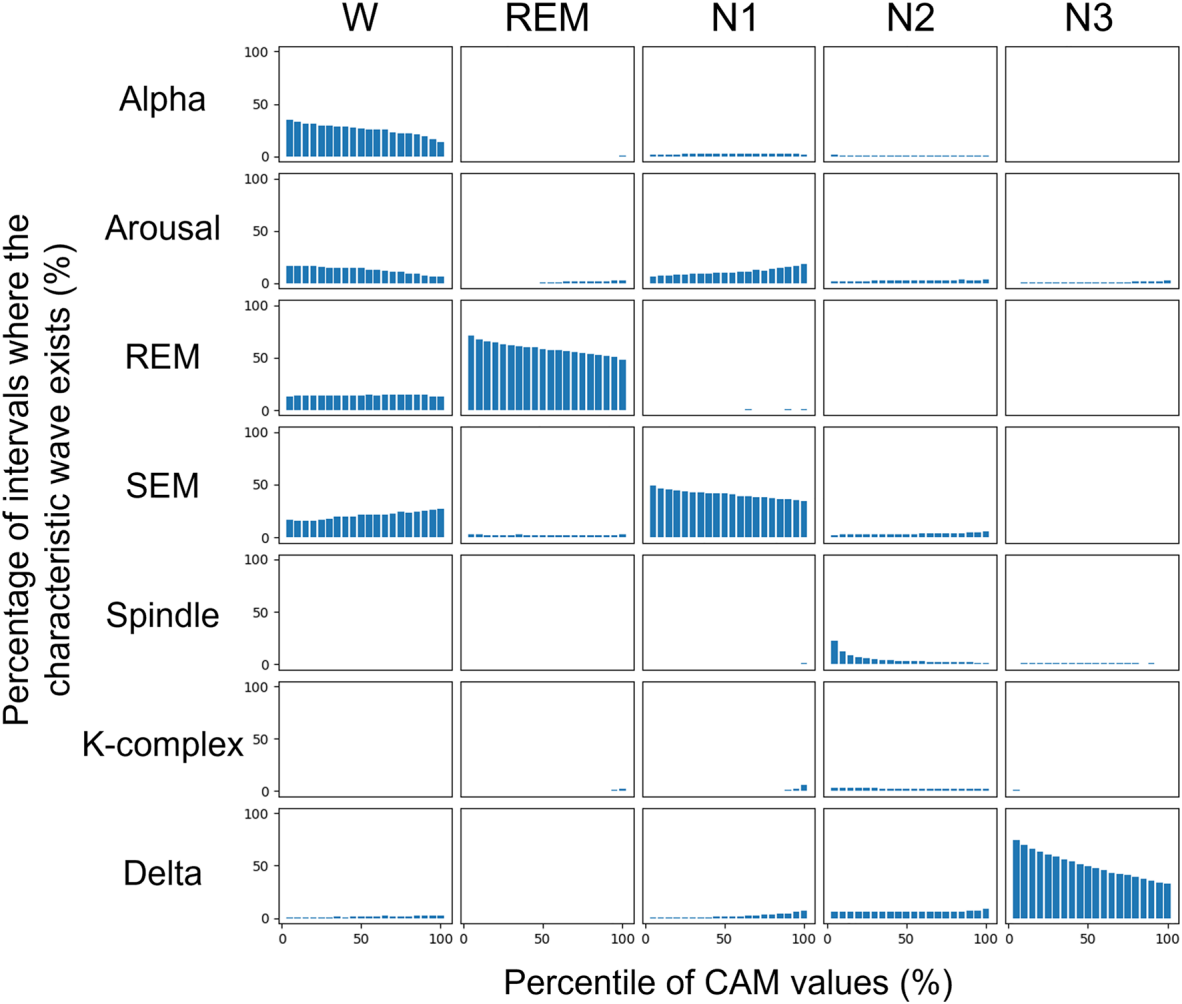
The relationship between the CAM values and the existence of characteristic waves in each sleep stage. The percentiles were aggregated for each true positive epoch.

In this study, we verified the overlapping of the focused sub-intervals and the typical characteristic waveforms: alpha, arousal, REM, SEM, Spindle, K-complex, and delta. These waves are the most important clues in manual scoring. Figure 3 shows the histograms of characteristic waves in each sleep stage obtained from the whole-night records of three young-adult subjects. We applied the seven Sleep-CAM models used in the seven-fold cross-validation to these records and count up the sub-intervals in the unit of 0.5 s.

Please note that the horizontal axis shows bins of time points with the top n% percentile CAM values for each epoch, and the vertical axis shows the percentage of time points which overlap with the corresponding characteristic waves. If certain characteristic waves exist more in the sub-intervals where the CAM values for Stage X are high, it means that they are more significantly referenced in the stage assignment for X. In particular, if smaller (left-hand side) percentile bins are more populated, it implies that the characteristic waves are more well referenced.

Some kinds of characteristic waves frequently appear in the intervals with high CAM values: alpha (Stage Wake), REM (Stage REM), SEM (Stage N1), spindle (Stage N2), and delta (Stage N3). This result suggests that the proposed model located the reasonable features to identify the sleep stages. Besides, it also shows that the reasoning mechanism will be helpful to find the characteristic waves. It will reduce the inspection workload and earn the trust of sleep experts.

However, as we suggested above, several critical characteristic waves were not used in the automated scoring process. Especially, the K-complex waves were not detected even though it is evidence as significant as a spindle. This may be related to the differences in detection capability. More specifically, the K-complex wave has characteristics that are very similar to those of the delta-rhythm wave (i.e., Stage N3) in that they have the same frequency components. Although human experts can distinguish these waves by checking the length of the wave, the organization and structure of Sleep-CAM prevent the identification of this information. Thus, in the Sleep-CAM system, the K-complex wave does not work as strong evidence for the occurrence of N2.

In addition, the proposed model also has a problem in arousal detection. Although arousal is an important event related to Stages W and N1, it was not considered important than alpha waves and SEM. In general, arousal is one of the sleep events defined as changing the peak frequency of the brain wave that suggests shifts from the current sleep stages to lighter ones. The system has to consider the sleep stage transition to detect this event, and that is why our proposed model cannot handle the arousal correctly except for the arousals accompanied by body movements. Actually, arousal is a special event in sleep diagnosis. For example, the number of arousals is an important sleep variable to diagnose sleep apnea syndrome (SAS). Designing a new deep-learning structure to detect arousal will be an essential task in our future work.

In contrast, Sleep-CAM might locate the waveforms not/rarely used in manual scoring as effective features. Figure 3 shows some cases in which the CAM value is high without any characteristic waves. Besides, the proposed method can deal with the epoch, which can not be scored without considering the surrounding epoch. One example is “tonic REM,” which is a type of REM sleep in which eye rolling does not occur. Experts tend to identify this type of REM sleep by considering the sleep stage transition rules. We suspect that the Sleep-CAM monitors EMG signals to identify tonic REM: it is not a decisive evidence in the manual scoring, but it is known that the amplitude of the EMG signal present during REM sleep tends to be smaller than the corresponding EMG signals in other sleep stages (Table 1). We suggest that Sleep-CAM carefully uses these characteristic waves to determine the sleep stage for every single epoch with high accuracy, although they are not considered to be important in manual scoring.

### Performance evaluation

Although the GAP layer is essential for CAM calculation, it also makes it difficult for the stage scoring to follow some AASM scoring rules, such as considering the timing of the characteristic waves’ appearance. Besides, our model is designed so that it will not consider the surrounding epochs, unlike the manual scoring process. So, we verified that Sleep-CAM is accurate enough for clinical practice.

To train/test the models, we mainly used the dataset with 109 whole-night sleep records obtained from the different healthy individuals. This dataset is a combination of the MASS dataset^21^ and our original dataset. The MASS dataset is one of the most popular sleep cohorts in the automated sleep-stage scoring area and uses the same measurement channels as in this study. However, the number of samples is a little bit small for training deep-learning models, so we added 49 sleep records obtained from the individuals in their 20–30 s. Of course, Finally, the datasets contains more than 110,000 samples (sleep epochs). the environment and measurement equipment associated with these datasets were different; however, these effects are thought to be small, so we decided to ignore these differences here.

The age distribution of participants in the MASS dataset is biased. It contains the sleep records obtained from individuals in their 20 s and over 50 s. To verify the robustness against the difference in individuals’ ages, we also examined the accuracy using only the Mass dataset or only our original dataset.

**Figure 4:**
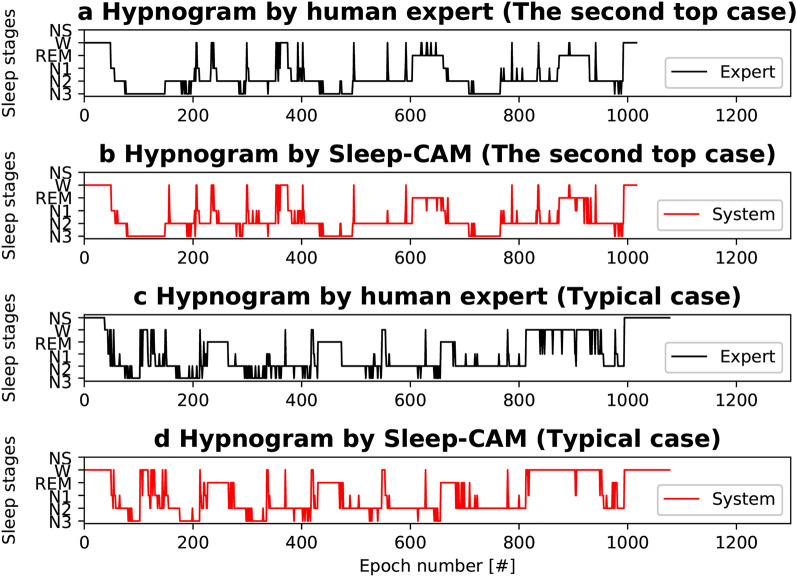
Hypnogram examples (second top case ${\mathrm{Acc.}}=93.8\%$ and typical case ${\mathrm{Acc.}}=86.8\%$). Sleep-CAM scored the same sleep stages as the expert in most epochs, except for those close to stage transitions. The epochs with “NS” were not scored due to noise. These epochs are ignored in the evaluation.

We randomly divided 109 sleep records into seven subsets of 17 records and conducted seven-fold cross-validation. Note that the performance indices were collectively calculated for all testing records across trial boundaries. These results show that our proposed model yielded a scoring accuracy and kappa statistics^22^ of $86.9\%$ and 0.81, respectively (Table 2a). Moreover, our model demonstrated achieving a sleep-stage score that is comparable to the inter-rater reliability among sleep experts ($82.0\%$)^23^. The kappa statistic results also suggest that the scoring results achieved by the proposed method are in almost perfect agreement with those of experts^22,24^. The comparison of hypnograms (Fig. 4a–d) also shows that the scoring results of Sleep-CAM are almost the same as those of the experts.

**Table 2:**
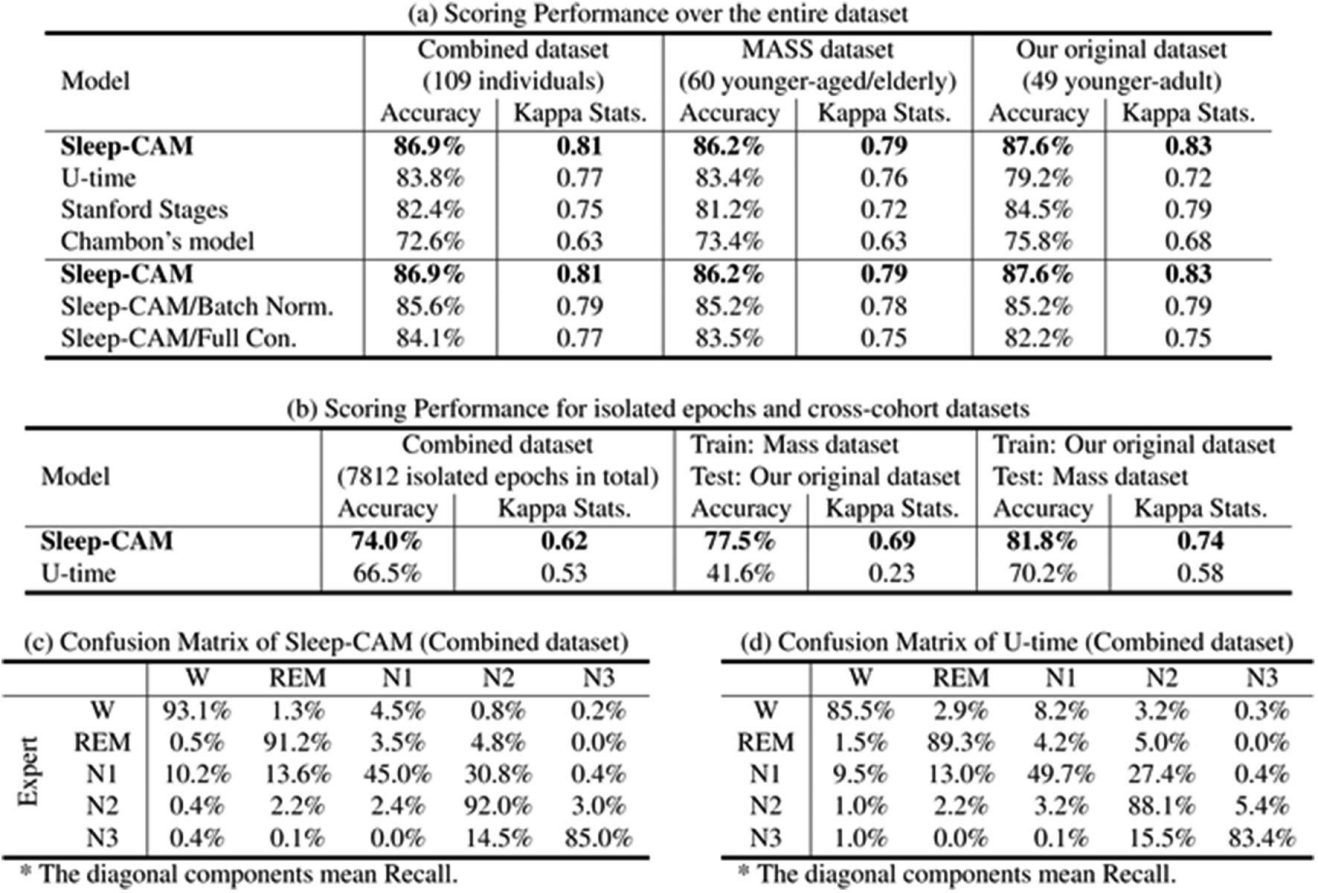
Comparison of scoring performance.

The diagonal components mean Recall.

Significant values are in bold.

However, the recall for Stage N1 is worse than the other sleep stages (Table 2c). The period of N1 sleep is found to be significantly shorter than those of other sleep stages. (In the case of our datasets, the ratio of N1 sleep was approximate $9.7\%$.) This unbalancing distribution of sleep stages might have worsened the N1 recall. Besides, according to the AASM rules, Stage N1 occurs independently of any characteristic waves. Specifically, it is the period between the disappearance of the alpha wave and the appearance of the K-complex/spindle wave. The proposed model is mainly designed to detect the characteristic waves, and it seems to be difficult to be aware of the importance of “disappearance” only with the signals in the target epoch. Using the signals in the surrounding epochs might lead to the improvement of the recall of N1.

In reality, we think that this problem will not be serious in practical usage. Stage N1 is known as the most difficult stage to score also in manual sleep-stage scoring. The kappa statistics for Stage N1 was 0.49, which was higher than that among the experts (0.35^25^. The Rechtschaffen & Kales method^26^ was used for scoring in their study^25^: however, the applied definition of N1 is not inconsistent with the corresponding AASM rule). Thus the poor recall for Stage N1 is commonly seen even in the manual scoring and will not lead to serious misdiagnosis.

**Figure 5:**
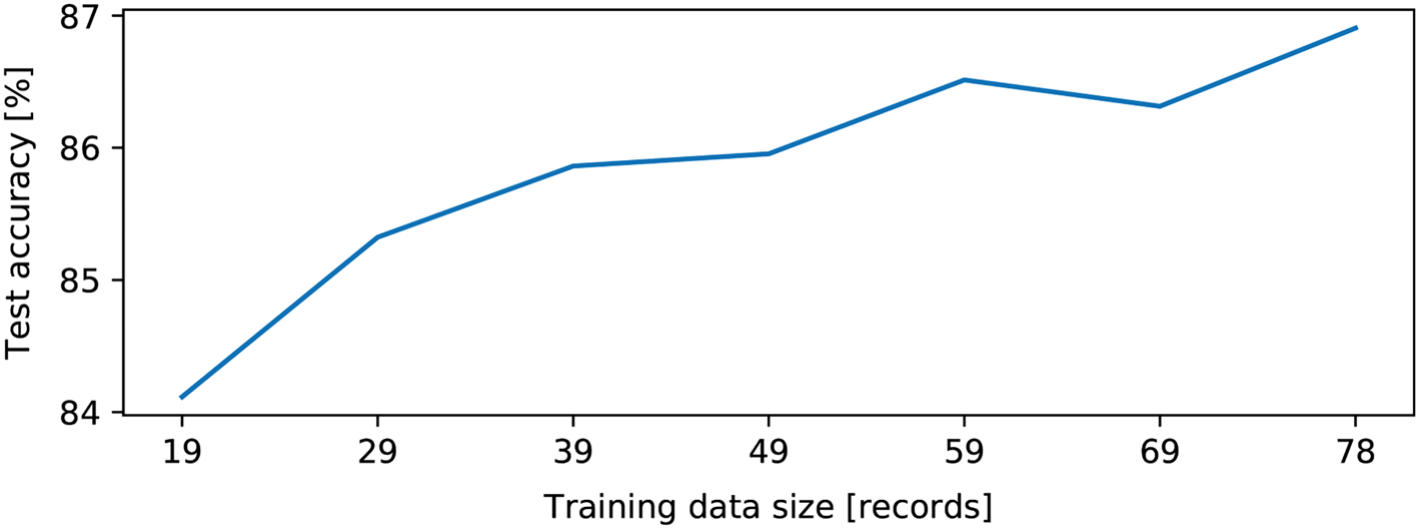
Change in scoring accuracy with the number of training samples.

Sleep-CAM also has some advantages in computing costs and sample preparation for training. The simplicity of the characteristic-wave detection module enables to reduce the computing costs. On a computer with sufficient specifications (GPU and 3GB Memory), the training process will take only about one hour and a half. Besides, the experiment also shows that it requires only 19 whole-night sleep records to achieve the scoring accuracy of 82.0% (Fig. 5), which is quite a small number for deep-learning models.

#### Comparison between GAP layer and full-connection layer

The evaluation experiment shows that Sleep-CAM is accurate enough for the clinical practice, even though it employs the GAP layer. The comparison between the proposed model and its “full-connection” counterpart (Table 2a) also indicates that the GAP layer leads to the improvement of the scoring accuracy. The comparison model has a full-connection layer with 128 neurons instead of convolutional and GAP layers in the assignment module. Even though the other conditions were the same as those of the proposed model, the full-connection model scored sleep stages with an accuracy of $84.1\%$ (2.8 points lower than that of Sleep-CAM, $p = 3.4 \times 10^{-7}$).

#### Efficacy of layer normalization

Although this is not the central topic of this study, we would like to mention the efficacy of layer normalization here. Compared to batch normalization, layer normalization is rarely employed in deep-learning models. However, we found that this normalization operation is effective for automated sleep stage scoring tasks.

To verify the efficacy of the layer normalization technique, we implemented a Sleep-CAM-like model, in which the only difference was that a batch normalization layer replaced the layer-normalization layer; we subsequently compared their scoring performance. The experimental results (Table 2a) revealed that the accuracy of the model with batch normalization tended to be approximately 1.3 points less than that of the proposed model ($p = 3.4 \times 10^{-7}$). These results suggest that layer normalization more effectively contributes to accurate characteristic wave detection.

This accuracy improvement seems to be due to the layer normalization process similar to several manual scoring operations. The AASM rules have defined the characteristic waves, such as K-complex and sleep spindle, according to “clearness,” i.e., the ratio of the amplitude of these waves to the background EEG signal. Such calculation, taking the relative values among the features, cannot be easily imitated by the combination of typical trainable layers.

In contrast, the layer normalization process is almost the same as this operation. Concretely, it normalizes the feature values such that a set of feature values at a certain time point follows a normal distribution with a respective mean and variance of 0 and 1. These relative feature values will help grasp whether the characteristic waves can be separated from the background brain waves, and this might be why Sleep-CAM achieved high scoring accuracy despite having fewer layers than other sleep-stage scoring models.

### Comparison with the existing models

We also compared the scoring accuracy of Sleep-CAM with those of the existing model: U-time^13^, Stanford Stages^2^ and Chambon’s model^3^.

The U-time is based on the U-net structure, which was originally designed for signal conversion tasks. It calculates the scoring result at each time point, like Sleep-CAM, which is why we chose it for comparison.

U-time requires 35-epoch signals for scoring; therefore, the scoring result at each time point is quite different from that of Sleep-CAM. It is affected not only by the existence of characteristic waves but also by the stages of surrounding epochs. So, the scoring result at each moment of U-time was relatively hard to interpret.

Stanford Stages and Chambon’s model have the version using only the signals in the target epoch as input. Then, we conducted the comparison experiment under the condition that the models can only use the information in the target epoch.

Please note that all hyper-parameters, settings, and preprocessing of these comparison models were optimized suitably for our sleep records. For example, we changed the first layer in U-time to be suitable for nine-channel inputs.

As compared to Sleep-CAM, the U-time scored the sleep stage with lower accuracy of 3.1 points. Note that *p* value of $1.2 \times 10^{-7}$ was obtained from the Wilcoxon signed-rank test with the Bonferroni adjustment method. Interestingly, the U-time model did not beat the proposed model, even though they used longer signals as input. Table 2d suggest that U-time has lower recall for each stages and U-time did not find effective features.

Besides, Table 2a shows that U-time could not achieve a stable scoring accuracy. For example, the accuracy of the U-time for our original dataset was 4.2 points lower than that for the MASS dataset, even though our original dataset has a narrow distribution of patients’ ages. Actually, we could not have a convincing reason, but we expected that the small number of training samples or the noise in the biological signals might affect the scoring performance. At least, it can be said that the condition in which U-time can demonstrate the high performance is comparatively narrower than that for Sleep-CAM.

U-time also seems to be weak against the difference in the training/testing dataset. The cross-cohort experiments (Table 2b), in which we used the different dataset during the training and testing steps, shows that the U-time were more affected in the scoring accuracy. Especially, the accuracy of U-time was only 41.6% when we trained it using the MASS dataset, then tested it with our original one. This result clearly shows that U-time did not locate the common features among different datasets.

Sleep-CAM was the most accurate model also among those using only the scoring-epoch signals as input. The Stanford stages and Chambon’s model scored sleep stages with accuracy of $82.4\%$, and $72.6\%$, respectively ($p = 5.1 \times 10^{-16}, 2.0 \times 10^{-16}$, for each).

## Discussion

### What characteristic waves were detected?

The most significant advantage of the proposed method is that it employs a reasoning mechanism. More specifically, this reasoning mechanism allows the experts to easily verify whether the scoring results are reasonable, which will result in the establishment of the experts’ trust.

Through the reasoning experiment, we found that alpha, REM, SEM, spindle, and delta waves were detected and were focused on in the scoring process. In contrast, the K-complex, arousal, and other characteristic waves were not detected and used actively. What is the cause of this difference?

These differences seem to derive mainly from how easy it is to find/detect in the training process. In the case of Sleep-CAM, the structure is not suitable for grasping the duration of the characteristic waves and the change in the peak frequency in EEG signals. These factors are essential to detect K-complex waves and arousals. Whether the model has a structure for detecting these characteristic waves well will be a key to achieving high scoring accuracy in the future.

### Impact of the GAP layer

As aforementioned, the experts have traditionally scored the sleep stages using time-related information, such as the timing and duration of the characteristic waves. In contrast, Sleep-CAM cannot utilize this criterion in the scoring procedure because of the GAP layer in the assignment module. It is natural to guess that the GAP layer may affect the scoring accuracy; however, we found that it was superior to the full-connection layer and is effective to improve the robustness against the change in the transition tendency of sleep stages (Table 2a).

The main reason is that the GAP layer reduces the number of trainable parameters in the assignment module. Consequently, this feature encourages more efficient model training, allows it to realize high performance, and prevents overfitting. Second, the GAP layer also allows the model to locate the features of characteristic waves quickly. For example, the model with a full-connection layer learns the characteristic waves appearing at different positions in the epoch as different features, even if these waves are of the same type, which is not desirable for our purpose. In comparison, the model with the GAP layer can extract these waves as one feature wherever the waves may appear in the epoch.

Despite such a positive point, the GAP layer may involve weakness. The GAP layer may cause decreasing the scoring accuracy for “isolated epochs,” an epoch whose sleep stage differs from its surrounding epochs. First, we expected the proposed model to achieve high accuracy for these isolated epochs because it ignores the signals in surrounding epochs. However, the experimental result (Table 2b, Fig. 4c, d) showed that it is not a complete solution for these isolated epochs.

In our understanding, the reason may be that these epochs often mix multiple sleep-stage periods and contain characteristic waves that correspond to multiple sleep stages. For example, N1 sleep stages with arousal, which often appear as isolated epochs, usually do not last one epoch. Besides, the stage transition often occurs in the middle of the epoch; these isolated epochs often contain multiple sleep-stage periods. As shown in Introduction, the experts score these epochs considering the percentage of the sleep-stage periods. The longest sleep stages will be assigned for these epochs. Whereas, Sleep-CAM cannot reproduce this procedure due to employing the GAP layer. As mentioned above, this layer integrates the class activation mapping along the time axis, and the information on how long each sleep stage lasts in the target epoch will be spoiled. Although the GAP layer works well in the reasoning process, it also decreases the scoring accuracy of isolated epochs.

Therefore, we guess that our model will not be good at scoring the SAS patients’ records because of the many arousals and isolated epochs. In clinical practice, the users have to check the property of the patients and decide whether the proposed model can be applicable or not. We would suggest that the gate and attention mechanism might effectively solve this problem without losing explainability but let us leave it as future work.

## Methods

All of the procedures were conducted in accordance with the Ethical Guidelines for Medical and Health Research Involving Human Subjects at the University of Tsukuba and the Declaration of Helsinki. Additionally, all procedures were approved by the Tsukuba Clinical Research and Development Organization (T-CReDO) of the University of Tsukuba (Approved ID# H29-177) and the Ethics Committee of the Center for Computational Sciences at the University of Tsukuba (Approved ID# 19-001). The study protocol (UMIN000029911) was registered with the University Hospital Medical Information Network Center. All the subjects are over 19 years old, and we obtained informed consent from all of them.

### Datasets

In this study, we mainly used three datasets: MASS dataset session 3, our original dataset (Table 3), and the combined dataset of these.

**Table 3:** Characteristics of the datasets.

Property		Original	MASS CS1 SS3
Participants	Number	49	60
	Ave. age ± S.D.	$24.0\pm 3.6$	$41.7 \pm 18.8$
	Male%	59.0	46.7
	# of SAS patients	4	0
	(Apnea Hypopnea Index $\ge 15$)		
Measurements	Filter (EEG)	Band-pass 0.3–35 Hz	Band-pass 0.3–100 Hz
		Notch 50 Hz	Notch 60 Hz
	Filter (EOG)	Band-pass 0.3–35 Hz	Band-pass 0.1–100 Hz
		Notch 50 Hz	Notch 60 Hz
	Filter (EMG)	Band-pass 10–100 Hz	Band-pass 10–100 Hz
		Notch 50 Hz	Notch 60 Hz
Contents	# of epochs	53,630	57,643
	Stage W%	19.8	10.8
	Stage REM%	13.6	17.8
	Stage N1%	9.7	8.2
	Stage N2%	42.8	50.1
	Stage N3%	14.0	13.1
Variables	Total sleep time (min)	$451.6\pm 54.1$	$456.3\pm 57.8$
	Efficiency (%)	80.7	89.7

The MASS dataset is an open-source sleep cohort with 60 records that were scored by one sleep expert. Two records (ID: 01-03-0034 and 01-03-0036) were excluded because of noise contamination. Additionally, most of the participants were healthy. We used this dataset to evaluate Sleep-CAM performance on the data of individuals in their 20 s and over 50 s. The PSG signals in this dataset were measured using sampling frequencies that differed from the Sleep-CAM standard. Thus, we applied FFT-based resampling to these signals. We used the version that we downloaded in September 2019.

Besides, we also used our original dataset, consisting of 49 records from different individuals in their 20 s and 30 s. All individuals were healthy, except for some SAS patients. In the measurement, PSG-1100 (Nihon Kohden, Tokyo, Japan) was used for obtaining six EEG signals (F3-M2, F4-M1, C3-M2, C4-M1, O2-M1, O1-M2), two EOG signals (E1-M2, E2-M2), and one EMG signal (EMG1-Z). The measurement setting and the position of the electrodes were according to AASM rules^1^. Besides, to make the signals more distinguishable, we calculated the respective differences between the EEG electrode potentials and their opposite-side mastoid electrodes. In some cases, we had to use different reference electrodes because of contact failure.

The sleep records were scored manually according to AASM rules^1^. One sleep expert provided the “correct” sleep-stage labels for an additional dataset. She achieved the intra-rater reliability of $92.1\%$, which suggests that her scoring results are reliable. Besides, many clinics cannot afford to use multiple experts for scoring a single record. In this sense, our scoring is consistent with the common environment in the clinical field.

In addition to these datasets, we also used three one-sleep-cycle records in the explainability evaluation. These records were obtained healthy individuals of in their 20 s (25+ to 2.1 years old), but are not included in our original dataset. To show where the characteristic waves appear/exist, the “characteristic-waves labels” were affixed by the expert. Please note that the labels have a time resolution of 0.5 s, and the labelled intervals are a little bit longer than the actual characteristic waves’ length.

In this paper, we focused on the seven characteristic waves: Alpha, Arousal, REM, SEM, Spindle, K-complex, and Delta. These characteristic waves are more reliable evidence than other characteristic waves shown in Table 1 for manual scoring process.

### Performance indices

To evaluate the scoring performance of Sleep-CAM, we used the overall accuracy and kappa statistics.

$$\begin{aligned}&{\mathrm{Accuracy}} = \frac{\sum _{s \in S}{{\mathrm{E}}_{({\mathrm{s}},{\mathrm{s}})}}}{{\mathrm{M}}}, \quad \quad \kappa = \frac{{\mathrm{Accuracy}}-p_e}{1-p_e}, \quad \quad p_e = \sum _{s \in S} \frac{\sum _{{\mathrm{u}} \in {\mathrm{S}}} {{\mathrm{E}}_{({\mathrm{s}},{\mathrm{u}})}}}{{\mathrm{M}}}\cdot \frac{\sum _{{\mathrm{u}} \in {\mathrm{S}}} {{\mathrm{E}}_{({\mathrm{u}},{\mathrm{s}})}}}{{\mathrm{M}}},\\&{{\mathrm{Recall}}_{s}} = \frac{{{\mathrm{E}}_{({\mathrm{s}},{\mathrm{s}})}}}{\sum _{u \in S}{{\mathrm{E}}_{({\mathrm{s}},{\mathrm{u}})}}}, \quad \quad {{\mathrm{Precision}}_{s}} = \frac{{{\mathrm{E}}_{({\mathrm{s}},{\mathrm{s}})}}}{\sum _{u \in S}{{\mathrm{E}}_{({\mathrm{u}},{\mathrm{s}})}}}, \\&(S = \{{\mathrm{W}}, {\mathrm{REM}}, {\mathrm{N1}}, {\mathrm{N2}}, {\mathrm{N3}}\}), \end{aligned}$$

where $E_{(s,u)}$ is the number of epochs scored as Stages *s* and *u* by the human expert and Sleep-CAM, respectively, and M is the total number of epochs used for testing. As mentioned above, we assumed that the human expert always provides the correct sleep-stage label. Thus, the accuracy was defined as being equal to the inter-rater reliability between the expert and proposed model.

The kappa statistic was used to evaluate the scoring agreement between the expert and Sleep-CAM. Unlike simple accuracy calculations, kappa statistics account for differences in the distribution of each sleep stage^22^. Generally, if the kappa statistic exceeds 0.8, the two results are considered to be in perfect agreement^22,24^.

As aforementioned, we calculated the accuracy and kappa statistics for all testing records across trial boundaries collectively because of the difference in the number of testing epochs for each trial.

### Computer hardware

We used three computers for the experiments using the workstation with following hardware components:

- $$<{{\textbf {Workstation 1}}}>$$
- **CPU** Intel Xeon Gold 5122 @ 3.5 GHz * 4
- **GPU** NVIDIA Quadro P6000 PCIe 24 GB * 2
- **Memory** 185 GB
- **OS** Ubuntu 18.04.3 LTS
- $$<{{\textbf {Workstation 2}}}>$$
- **CPU** Intel Xeon Platinum 8180 @ 2.5 GHz * 56
- **GPU** NVIDIA Tesla V100 PCIe 32 GB * 4
- **Memory** 1.5 TB
- **OS** Ubuntu 18.04.3 LTS
- $$<{{\textbf {Workstation 3}}}>$$
- **CPU** Intel Xeon Gold 6154 @ 3.0 GHz * 36
- **GPU** NVIDIA Tesla V100 PCIe 32 GB * 8
- **Memory** 750 GB
- **OS** Ubuntu 18.04.5 LTS

To implement Sleep-CAM and conduct the experiments, we used Python 3.6.9, TensorFlow-GPU 2.0.3/2.1.0^27^, and Keras^28^. We also used Cygnus^29^ in some preliminary experiments; it is a high-performance computer managed by the Center for Computational Sciences at the University of Tsukuba.

## Related work

### Deep learning models for sleep stage scoring

To improve the efficiency of sleep stage scoring, many researchers and research groups have developed automated sleep-stage scoring methods. Especially, the deep learning technology made a great contribution in improving the scoring accuracy^2–10,13^. Our research is also one of the development research of the stage-scoring methods using the deep neural networks.

However, we think that our development policy is quite different from those of the existing study^2–10,13^. Concretely, we regard the proposed models as one of the tools for daily sleep diagnosis by human experts; in contrast, the existing models appear to be “alternatives” to the manual scoring. In other words, the existing methods do not assume experts to intervene in the scoring process.

For example, many models do not use the PSG signals to avoid the difficulty of handling many types of biological signals simultaneously and reduce the workload for electrode placement^4–10,12,13^. Specifically, DeepSleepNet^4^, SleepEEGNet^5^, the model developed by Tsinalis et al.^6^, and U-time^13^ focus on a single EEG signal. Other methods^12^ score sleep stages based on the electrocardiogram signal and respiration. This improvement makes the model simple and the scoring more accurate. However, there is no manual scoring rule for these signals; so, the experts cannot verify whether the automated scoring results are correct.

Lacking reasoning mechanism^2–12^ also prevents the experts’ interaction. The experts have to score the sleep stage by themselves to grasp the characteristics of the scoring epochs. Therefore, if the experts want to reduce their workloads by these existing models, they must trust their scoring results even though these models do not provide information to gain credibility.

In this paper, we used three existing models for comparison: U-time^13^, Stanford Stages^2^, and the Chambon’s model^3^. Here, we will explain these models.

### U-time

U-time is a unique scoring model based on a signal conversion model, U-Net. Like other signal conversion models, U-Net consists of an encoder and a decoder. The encoder compresses the signals and extracts the features, while the decoder decompresses the extracted features and makes the converted signals. Especially, the U-net is characterized by “skip connection.” The encoder provides the low-order features extracted in the early layer to the decoder; therefore, both local and global information can be used in the transformation process. This characteristic seems to be suitable for sleep stage scoring. This inspection needs to consider not only the local information such as characteristics waves but also the global information like sleep stage transition.

Besides, U-time calculates the stage likelihood map like CAM because the U-Net was originally designed as a signal conversion model. Unlike Sleep-CAM, it uses 35-epoch-length signals as input, so each likelihood value is determined by much longer signals. As a result, the likelihood map is a little bit difficult to understand: it reflects not only the existence of characteristic waves but also the sleep stage transition/tendency.

Unfortunately, its accuracy is not superior to the other methods such as DeepSleepNet^4^, despite its high computing amount. However, its explainability is clearly higher than other most existing methods with no reasoning mechanism. Thus, we think it is one of the most reliable scoring models except for Sleep-CAM.

### Stanford stages

Stanford Stages^2^ is a state-of-the-art model for sleep-stage scoring proposed by J.B. Stephansen et al. in 2018; it utilizes PSG signals and achieved a scoring accuracy of 87%. This model consists of three phases: preprocessing, VGG16^30^, and stage assignment.

Unlike our proposed method, the Stanford model calculates PSG signal autocorrelations and emphasizes the frequency-domain features. The VGG16-based component, which is a popular deep-learning model for general image recognition, identifies the sleep stages from the converted signals. Interestingly, this model splits the epoch into two or six sub-sequences and assigns one sleep-stage label for each sub-sequence. Finally, the stage assignment module integrates the sub-sequence stage labels and determines the sleep stage for an entire epoch. This structure is equivalent to the third principle for stage assignment described in the “Sleep stage scoring” section. The most significant disadvantage of this model is the computing time. Specifically, VGG16 is a model that employs many convolutional layers; this significantly increases the computational cost.

### Chambon’s model

In 2018, Chambon et al. proposed a deep-learning model for sleep-stage scoring that consists of characteristic-wave detection and stage assignment components^3^. Although the overall structure of their model is moderately similar to that of our proposed model, each component is very different. Specifically, their model has two CNN blocks for wave detection, where one CNN block is used for EEG and EOG signals, and the other is utilized for an EMG signal. In addition, their model does not include layers that we found to be significant, such as layer-normalization and global average pooling layers. Because of these differences, the accuracy of Chambon’s model was consistently lower than that of our model.

## Conclusion

In this paper, we proposed a novel sleep-stage scoring model, Sleep-CAM, with a reasoning mechanism and verified its performance and what characteristic waves are detected/focused in the scoring process.

The experiment showed that the proposed model has the ability to reveal which sub-intervals were taken into account in the scoring decision. This allows the experts to easily verify whether the scoring procedure was reasonable.

In the characteristic wave analysis, we verified that the proposed model extracted alpha, REM, SEM, spindle, and delta waves and used them as strong evidences/clues for scoring each sleep stage. These characteristic waves are also significant in the manual sleep-stage scoring process. This result will prove to be evidence that the proposed model can be trusted. In addition, we also discussed the relationship between the model structure and the characteristic waveforms. It will be helpful to design a new automated scoring model.

Besides, The performance evaluation confirmed that our proposed method achieved scoring accuracies of $86.9\%$; these values are higher than the experts’ inter-rater reliability ($82.0\%$)^23^. We also verified that our model outperformed the existing models in terms of accuracy, and the scoring results were less affected by the age group of independents.

We believe that our model has sufficient potential for practical clinical use. But, we also think that some limitations need to be alleviated in order to use it in the actual clinical environment. For example, the low scoring accuracy for the isolated epochs will be a critical issue. The individuals with isolated epochs, such as the elderly and the SAS patients, are common in sleep practice. Besides, the detection of the arousal and K-complex will also need to be addressed.

At least, our study provided some novel findings that will be useful in developing automated sleep-stage scoring models. We hope that our study will contribute to realizing the world where everyone can overcome their sleep problems.

## Supplementary Information

Supplementary Information.

## Data Availability

The datasets analyzed during the current study are available from the corresponding author on reasonable request.
