# Supplementary material for: Automated sleep stage scoring employing a reasoning mechanism and evaluation of its explainability
Source: Sci Rep. 2022 Jul 27;12:12799. doi: 10.1038/s41598-022-16334-9 (PMC9329306; doi:10.1038/s41598-022-16334-9)
Supplement: Supplementary file 1 — Supplementary Information. [file 41598_2022_16334_MOESM1_ESM.pdf]

Supplementary Information

Preliminary Experiments for designing Sleep-CAM

Sleep-CAM does not consider the surrounding epochs, unlike manual scoring. As mentioned in the paper body, the GAP layer makes it difficult to use the signals in surrounding epochs without a more complicated assignment module. This chapter shows the scoring accuracy of the Sleep-CAM model combining the signals in the target epochs and the latter half of the previous epoch. Please note that it increases the length of input signals but does not have any additional structure/layer.

In this experiment, we used the 59 whole-night sleep records obtained by PSG-1100. The dataset was slightly different from "our original dataset" in the paper body and contained multiple sleep records from one participant. Besides, the condition for stopping training process was also different, and the number of training iteration was about twice as many as that in the paper. These differences affected the accuracy of Sleep-CAM in the following table.

Table A. 1. Scoring accuracy comparison (63326 epochs, 4-fold cross validation).

	Accuracy	Kappa Statistics
Sleep-CAM	88.2 %	0.84
+ longer input signal	88.1 %	0.83

The experimental result (Table A. 1.) suggests that the signals in the latter half of the previous epoch cannot improve the scoring accuracy. In our understanding, this is caused by the difference in the scoring process. In the manual sleep stage scoring, the signals in the latter half of the previous epoch are considered only under some conditions, such as cases that there are no characteristic waves in the current epoch. However, the proposed model has no mechanism to switch the process, and the latter half of the previous epoch is always considered.

Besides, the GAP layer loses the timing information when the characteristic waves appear. The model cannot separate the characteristic waves in the current epoch and the previous epoch. These differences would make it difficult to use the previous epoch effectively.

Of course, it would be possible to make such a mechanism to switch the process, but we have made the assignment module and reasoning mechanism simple in this research.
